# Supplementary material for: Jagged-mediated lateral induction patterns Notch3 signaling within adult neural stem cell populations
Source: Nat Commun. 2026 Mar 14;17:3986. doi: 10.1038/s41467-026-70478-0 (PMC13136484; doi:10.1038/s41467-026-70478-0)
Supplement: Supplementary file 1 — Supplementary Information [file 41467_2026_70478_MOESM1_ESM.pdf]

Jagged-mediated lateral induction patterns Notch3 signaling within adult neural stem cell populations

Sara Ortica, Miguel Martinez Herrera, Louis Degroux, Bastian Rochette, Nicolas Dray and Laure Bally-Cuif

Supplementary information

This file contains :

- **Supplementary Figures and Legends**
- **Supplementary Table 1**
- **Supplementary References**

Supplementary Figures and Legends

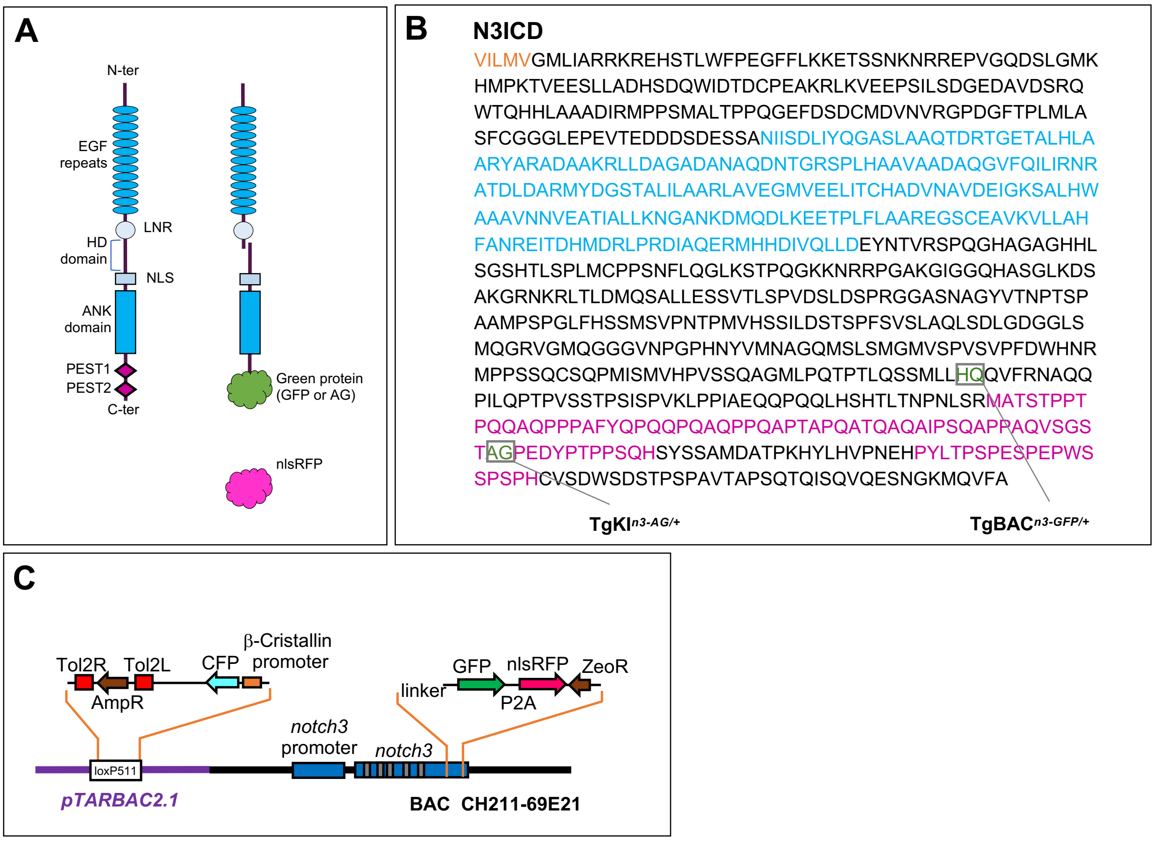

D

	Sequence	Spec. Score	Eff. Score	Off-targets	Off-targets +PAM
**gRNA2**	GGAGCTGTGACAGCTGGGCTGGG	94	63	0-0-0-2-35	0-0-0-0-1
**gRNA4**	GGGGTAATCCTCTGGGCCTGCGG	99	77	0-0-0-0-3	0-0-0-0-0

(28911)CTGCCTCCCATTGCTGAGCAGCAGCCGCAGCAACTTCACAGCCACACCCTCACCAACCCCAACCTCTCCCGCATGG
CCACCTCCACTCCCCCAACTCCACAGCAGGCACAGCCTCCTCCAGCTTTCTACCAACCACAACAGCCACAAGCTCAGCCGC
CGCAGGCCCCGACAGCCCCTCAGGCAACGCAAGCACAAGCTATCCCATCTCAGGCACCCCCAGCACAGGTGAGCGGCAG
CA**CCGCAGGCCCAGAGGATTACCCC**ACACCACCTTCCCAGCACAGCTACAGCTCAGCCATGGATGCAACGCCTAAGCATTA
TCTGCATGTGCCCAATGAACATCCTTACCTGACCCCTTCTCCAGAGTCTCCAGAGCCCTGGTCCAGCCCTTCCCCTCACTGT
GTGTCTGATTGGTCTGACTCCACA**CCCAGCCCAGCTGTCACAGCTCC**TTCTCAAACCCAGATTTCACAAGTCCAGGAGTCCA
ACGGGAAGATGCAGGTGTTTGCT***TGA***GCAGCCCAACCTACCTTAAAT***(29438)***

E

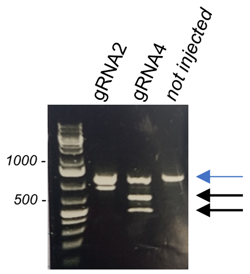

F

	Efficiency	Founders	Transmission rate
**Injection #1**	3,3%	1/60	3,3%
**Injection #2**	2,8%	1/36	20%

Figure S1. Details on the BAC and Crispr recombination procedures. A. Left: schema of the Notch3 protein with some regions of interest: Epidermal Growth Factor (EGF) repeats, Lin-12/Notch repeat (LNR) and Heterodimerization (HD) domain on the extracellular domain, Nuclear Localization Signals (NLS), Ankyrin repeats (ANK) domain and two Proline/glutamic acid/serine/threonine rich domains (PEST1/2) in the intracellular domain. Right: scheme of the Notch3 fusion proteins expected after transgenesis: a green fluorescent protein (GFP or Azami Green -AG-) followed by a ribosome skipping P2A domain and a nuclear RFP (nlsRFP, magenta) are inserted at the C-terminus, partially or totally disrupting Notch3 PEST domains. **B.** Aminoacidic sequence of the zebrafish Notch3 intracellular

domain (N3ICD). Orange: putative gamma-secretase cleavage site; blue: ANK domain; magenta: PEST domains; green: sites of insertion of the fluorescent reporter proteins for the TgBAC$^{n3\text{-}GFP/+}$ and the TgKI$^{n3\text{-}AG/+}$ lines. **C.** Scheme of modifications performed on BAC CH211-69E21 carrying the zebrafish *notch3* promoter and gene sequence, used to generate the TgBAC$^{n3\text{-}GFP/+}$ line. First, the *pTARBAC2.1* vector was modified at the *loxP511* site to insert a CFP under the beta-crystallin promoter as a transgenesis marker, as well as the iTol2-Amp cassette for random integration in zebrafish genome and Ampicillin selection. Then, a cassette containing the GFP-P2A-nlsRFP sequence was inserted in frame into *notch3* exon33. Removal of the Zeocine Resistance (ZeoR) cassette used for selection could not be performed. **D.** Top: guideRNA (gRNA) sequences tested to generate the TgKI$^{n3\text{-}AG/+}$ line; and their scores as noted on the CRISPOR web tool [1] (specificity score, predicted efficiency according to Moreno-Mateos parameters [2], number of off-targets for 0-1-2-3-4 mismatches and number of off-targets followed by a PAM for 0-1-2-3-4 mismatches). Bottom: position of gRNA2 and gRNA4 (in green) relative to PEST sequences (magenta) and STOP codon (red) of the *notch3* gene (start and end positions relative to [NCBI Reference Sequence: NC_007114.7] are indicated). **E.** A T7 Endonuclease1 test was performed on PCRs obtained from pools of 20 embryos injected with gRNA2, gRNA4 or not injected. Endonuclease1 cleaves only not-perfectly matched DNA, as it results from CRISPR/Cas9-generated indels. Both gRNA induce indels, but gRNA4 shows a higher percentage of cut (two fragments – black arrows) over uncut (blue arrow) DNA (estimated to 50%), and was selected to generate the TgKI$^{n3\text{-}AG/+}$ line. **F.** Two rounds of injections in *casper* fish were performed using gRNA4, Cas9 protein and donor vector as in panel B. Percentages of knock-in efficiency and transmission to the first generation are indicated.

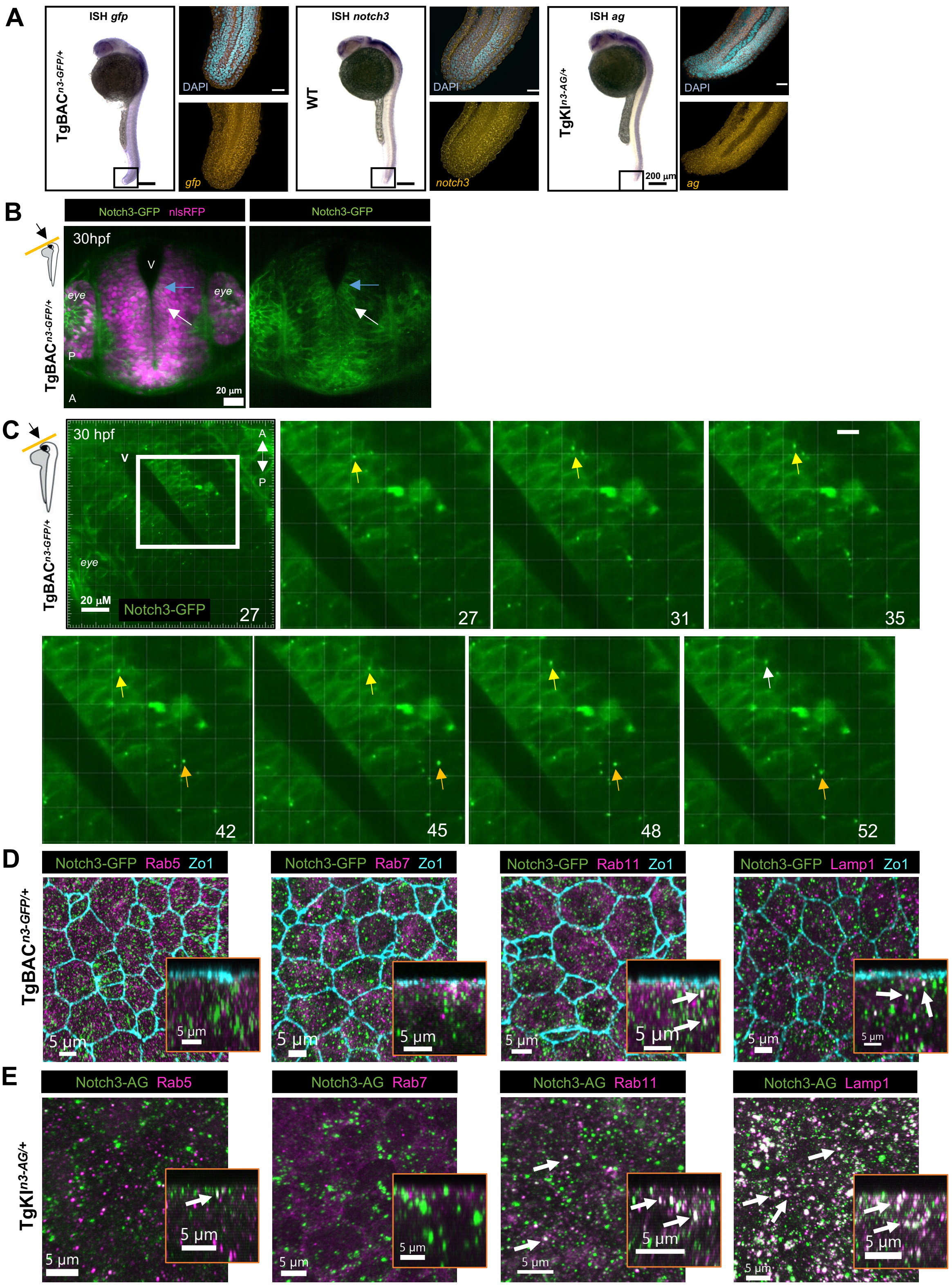

<u>Figure S2.</u> Notch3 fusion alleles report the different subcellular localizations of the Notch3 protein or protein fragments and their intracellular trafficking. A. Left panels: chromogenic in situ hybridization (ISH) on 28 hours post fertilization (hpf) embryos showing the expression patterns of *gfp* and *AzamiGreen* (*ag*) compared to *notch3* for each genotype-probe combination. Scale bars: 200 µm. Right panels: fluorescent in situ hybridization on 28 hours post fertilization (hpf) embryos with the *gfp*, *ag* and *notch3* probes. Spinning-disk single Z-section images of the posterior trunk region (similar location

indicated on embryos in left panels). Expression (orange dots) in all cases is excluded from the notochord, as expected. Scale bars: 30 μm. **B**. Left: schematic of the Light sheet microscope imaging angle of a 30hpf TgBAC$^{n3\text{-}GFP/+}$ embryo. Right: snapshot of an optical cross section across the embryonic forebrain, with Notch3-GFP in green and nlsRFP in magenta (V = Ventricle, A-P = Anterior-Posterior axis). Scale bar: 20 μm. The white arrow indicates Notch3-GFP at the membrane and the blue arrow indicates GFP nuclear signal. **C.** Snapshots of a short confocal movie of a 30hpf TgBAC$^{n3\text{-}GFP/+}$ living embryo (Notch3-AG in green). The first image illustrates the whole forebrain region that was recorded, and following images are magnifications of the indicated area (white square) at different times points (time in seconds indicated at the bottom of each image). V = Ventricle, A-P = Anterior-Posterior axis. Scale bar: 20 μm. Yellow and orange arrows show two moving green dots. **D,E.** Whole-mount dorsal confocal views of the adult pallial ventricular zone in each transgenic line, immunostained for endocytic vesicles markers: early endosomes: Rab5, late endosomes: Rab7, recycling endosomes: Rab11, and lysosomes: Lamp1. For each image, a detail of an orthogonal section at higher magnification is shown in inset. White arrows indicate colocalization of Notch3-fusion proteins (green) and endocytic vesicle markers (magenta) (Zo1 in cyan). The apparently different distribution behavior of Notch-GFP (absent in sorting endosomes) and Notch3-AG remains to be interpreted. GFP is reversibly quenched at acidic pH, which has led to differential tracking of GFP versus mCherry fluorophores in Drosophila [3,4]. IHC will be sensitive if quenching is associated with a denatured protein structure. More resolutive imaging would also be needed. Scale bars: 5 μm.

Representative images from at least two independent experiments are shown.

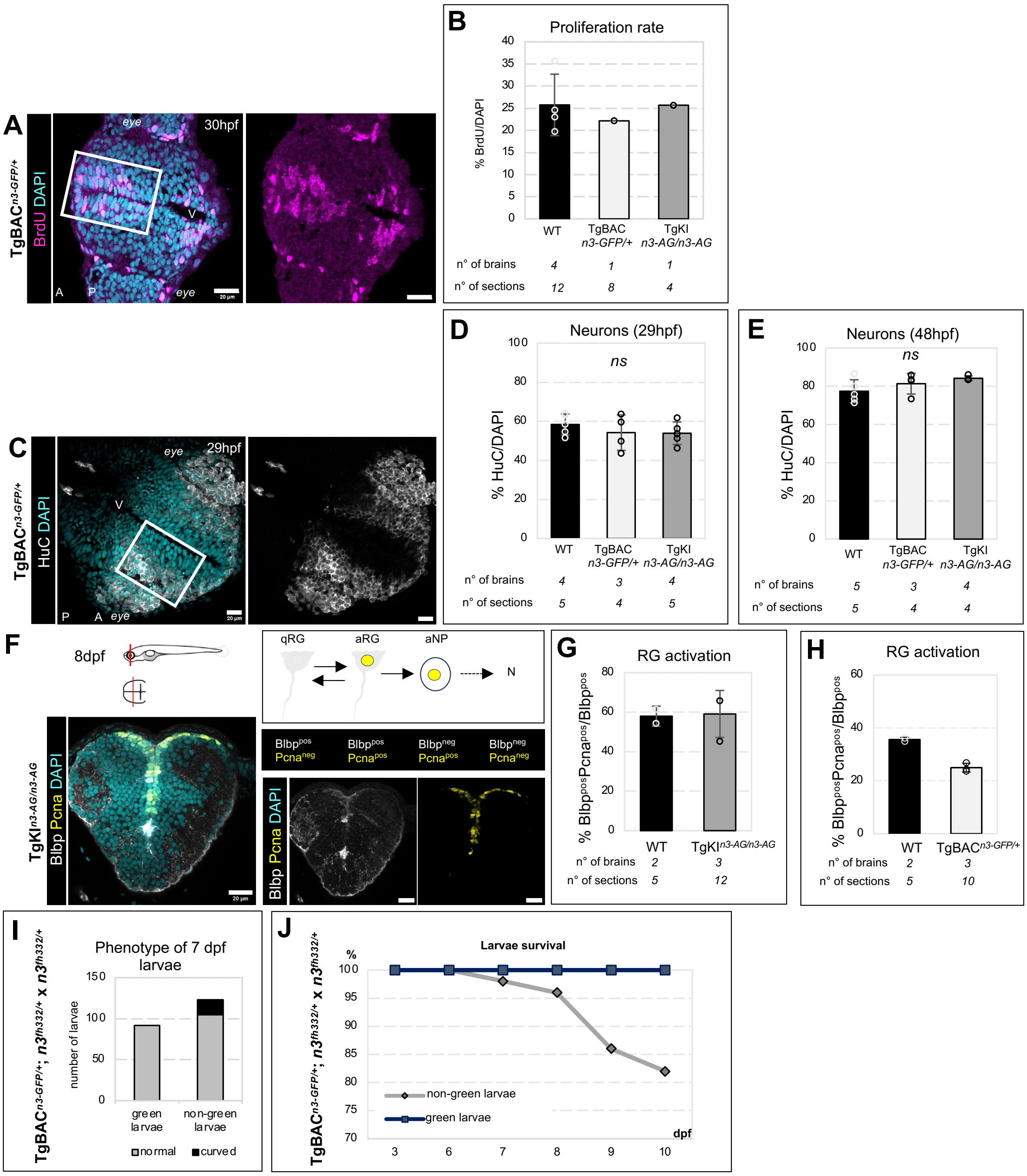

Figure S3. Functionality of Notch3 fusion alleles in embryos and larvae. A,B. A 20-minute BrdU pulse was applied to WT, TgBAC$^{n3\text{-}GFP/+}$ and TgKI$^{n3\text{-}AG/n3\text{-}AG}$ embryos at 30hpf. **A.** Forebrain cross sections (10 µm-thick) immunostained for BrdU (magenta) and counterstained with DAPI (cyan) (V = ventricle, A-P = Anterior-Posterior axis). Scale 20 µm. **B**. Proliferation rates (percentage of BrdU^{pos} cells in the area boxed in white in A) between genotypes. **C-E.** Quantification of neuronal production in WT, TgBAC$^{n3\text{-}GFP/+}$ and TgKI$^{n3\text{-}AG/n3\text{-}AG}$ embryos at 29 and 48hpf. **C**. Forebrain cross sections (10 µm-thick) at 29hpf immunostained for HuC (white) and counterstained with DAPI (cyan) (V = ventricle, A-P = Anterior-Posterior axis). Scale 20 µm. The same region was compared between brains (white square) to evaluate neuronal production at 29hpf and 48hpf. **D,E**. Neuronal production (percentage of HuC^{pos} cells among all cells in the area boxed in white in (C) -or an equivalent area at 48hpf-) between genotypes (Welch's ANOVA test, p-value=0.48 at 19hpf and 0.16 at 48hpf, ns: non-significant). **F-H.** Quantification of radial glia (RG) activation in WT, TgBAC$^{n3\text{-}GFP/+}$ and TgKI$^{n3\text{-}AG/n3\text{-}AG}$ larval pallia at 8dpf. **F.** Images: left: example

of a 50 µm-thick vibratome section across the telencephalon immunostained for Blbp (white) and Pcna (yellow) and counterstained with DAPI; middle and right: individual fluorescence channels. RG: $Blbp^{pos}$ (white); neural progenitors (NP): $Blbp^{neg}$; proliferating cell: $Pcna^{pos}$ (yellow). Scheme: schematic lineage progression depicting quiescent (q) and activated (a) RG, NPs, and neurons (N). Activated cells depicted with a yellow nucleus. Scale bar: 20 µm. **G,H.** Percentages of activated RG ($Blbp^{pos}$;$Pcna^{pos}$ among $Blbp^{pos}$ cells) compared between WT and $TgKI^{n3\text{-}AG/n3\text{-}AG}$ (**G**) (Wilcoxon text, p-value=0.8, ns), or WT and $TgBAC^{n3\text{-}GFP/+}$ (**H**) (Wilcoxon test, p-value=0.1066). **I.** Morphological phenotype of 7dpf larvae from a cross between a $TgBAC^{n3\text{-}GFP/+}$; $n3^{fh332/+}$ fish and a $n3^{fh332/+}$ fish. GFP^{pos} larvae ("green") appear normal, while a measurable proportion of GFP^{neg} larvae ("non-green") display a curved phenotype at 7 dpf, similar to $n3^{fh332/fh332}$ larvae. **J.** Percentage of surviving larvae resulting from a cross between a $TgBAC^{n3\text{-}GFP/+}$ $n3^{fh332/+}$ fish with a $n3^{fh332/+}$ fish. $n3^{fh332/fh332}$ larvae die between 6dpf and 10dpf (grey line). The presence of the fluorescent $TgBAC^{n3\text{-}GFP/+}$ copy can completely rescue the mortality (blue line).

Bar plots (B, D, E, G, H), show data as mean values +/- SD, with individual hemisphere values as dots; n numbers are indicated below each bar. All source data are provided as a Source Data file.

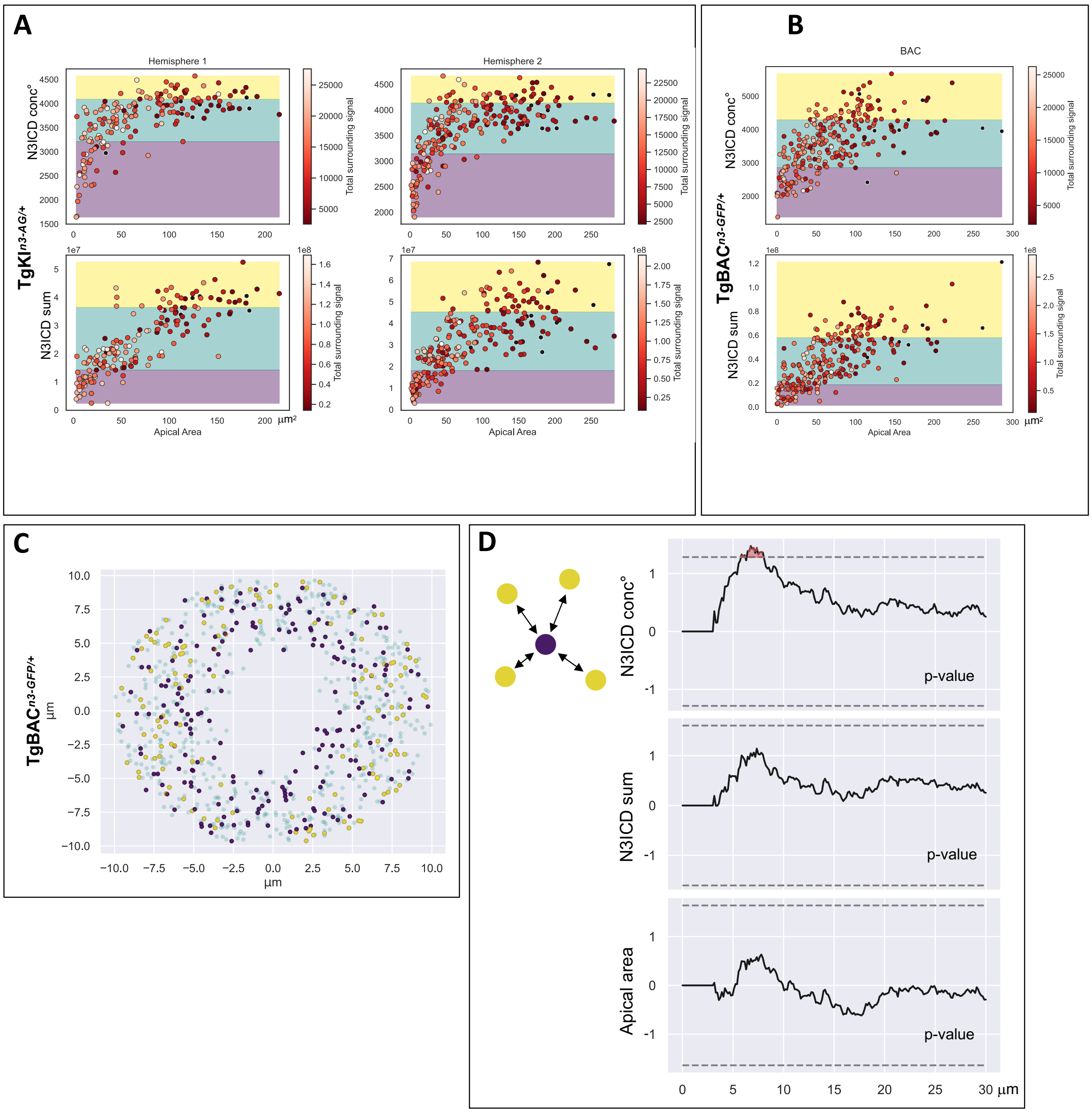

<u>Figure S4.</u> Spatial analysis of N3ICD levels in link with apical area size and in TgBAC$^{n3\text{-}GFP}$ fish. A,B. Scatter plots of N3ICD levels (y axis, normalized to nuclear volume - conc°- or in absolute values -sum-) relative to apical area size (x axis, in µm^2) in two pallial hemispheres of a TgKI$^{n3\text{-}AG/+}$ fish (same as Fig.3) (**A**) and one pallial hemisphere if a TgBAC$^{n3\text{-}GFP/+}$ fish (**B**). Each dot represents a cell, and background colors (purple, green, yellow) are the same color-coded cutoffs as in Fig.3. Red shades inside the dots measure N3ICD levels summed in the immediate neighborhood of each cell. **C.** Superposed neighborhoods of all N3ICD^{low} cells in a TgBAC$^{n3\text{-}GFP/+}$ fish. For each cell in this group, all the cells surrounding it at a maximal radius of 10µm are plotted at their position with respect to the original cell, color-coded as in B. **D.** Deviation from marked L-functions from an uncorrelated model as a function of the distance (radius in micrometers, x axis) from cells of low N3ICD values (scheme on left). Identity of the mark from top to bottom: N3ICD conc° per nucleus, N3ICD sum per nucleus, apical area. n=256 cells from 1 brain (2 hemispheres). Dashed lines indicate the hypothesis rejection threshold. p-values: N3ICD conc°: 0.01, N3ICD sum: 0.08, apical area: 0.85.

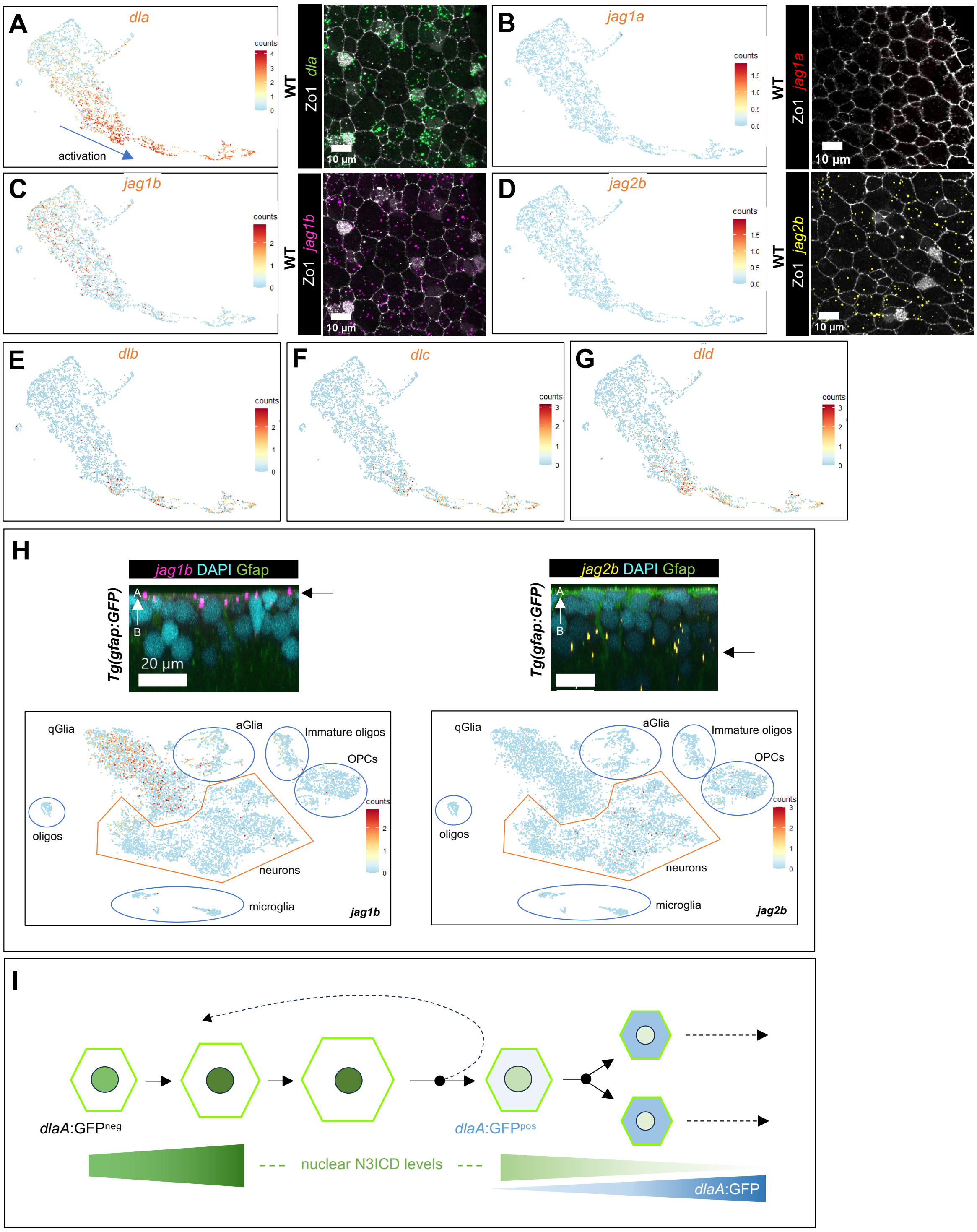

Figure S5. **Expression of Notch ligands and interpretation of Notch3 signaling levels in the adult pallial NSC population. A-G.** Expression levels of the different Notch ligand genes, plotted on the scRNAseq UMAP of neural stem and progenitor cells of the adult pallium in WT fish (left) [5], and revealed by smFISH (green, magenta or yellow) together with IHC for Pcna and Zo1 (white) on whole-mount confocal dorsal views of adult pallia (right). Scale 10 µm. **H.** Top: comparison of *jag1b* (magenta, left) and *jag2b* (yellow, right) expression revealed by smFISH on *Tg(gfap:GFP)* adult pallia together with IHC for GFP (green) and DAPI counterstaining (orthogonal sections of the ventricular zone). Note the apical localization of *jag1b* transcripts (in NSC, GFAP^{pos} cells) and the deeper localization of *jag2b* transcripts (mainly in GFAP^{neg} cells). Scale bar: 20 µm (A-B = apicobasal axis). Bottom: distribution of

jag1b (left) and *jag2b* (right) expression on scRNAseq UMAP of all cells of a WT adult pallium (with cell types indicated [5]). *jag1b* expression is mainly found in the RG population, while *jag2b* is weakly expressed in RG and rather expressed in neurons. **I.** Interpretative scheme of the variations of nuclear N3ICD levels in NSCs during quiescence and as they progress along the lineage (over time from left to right). NSCs (hexagons) are represented from their apical surface, in scale, with a green plasma membrane (Notch3-fluo). Green shades in cell nuclei (circle in center of hexagons) and graded triangles below reflect nuclear N3ICD levels. *dla*^{neg} and *dla*^{pos} NSCs are shown in white and blue, respectively, which progressively higher *dla*:GFP intensities along lineage progression. Cell division events are indicated by black dots, they are separated by phases of quiescence. Lineage progression is drawn from [6], and N3ICD levels are based on Fig.4. Of note, they were analyzed at the population level and not along an individual lineage as depicted here.

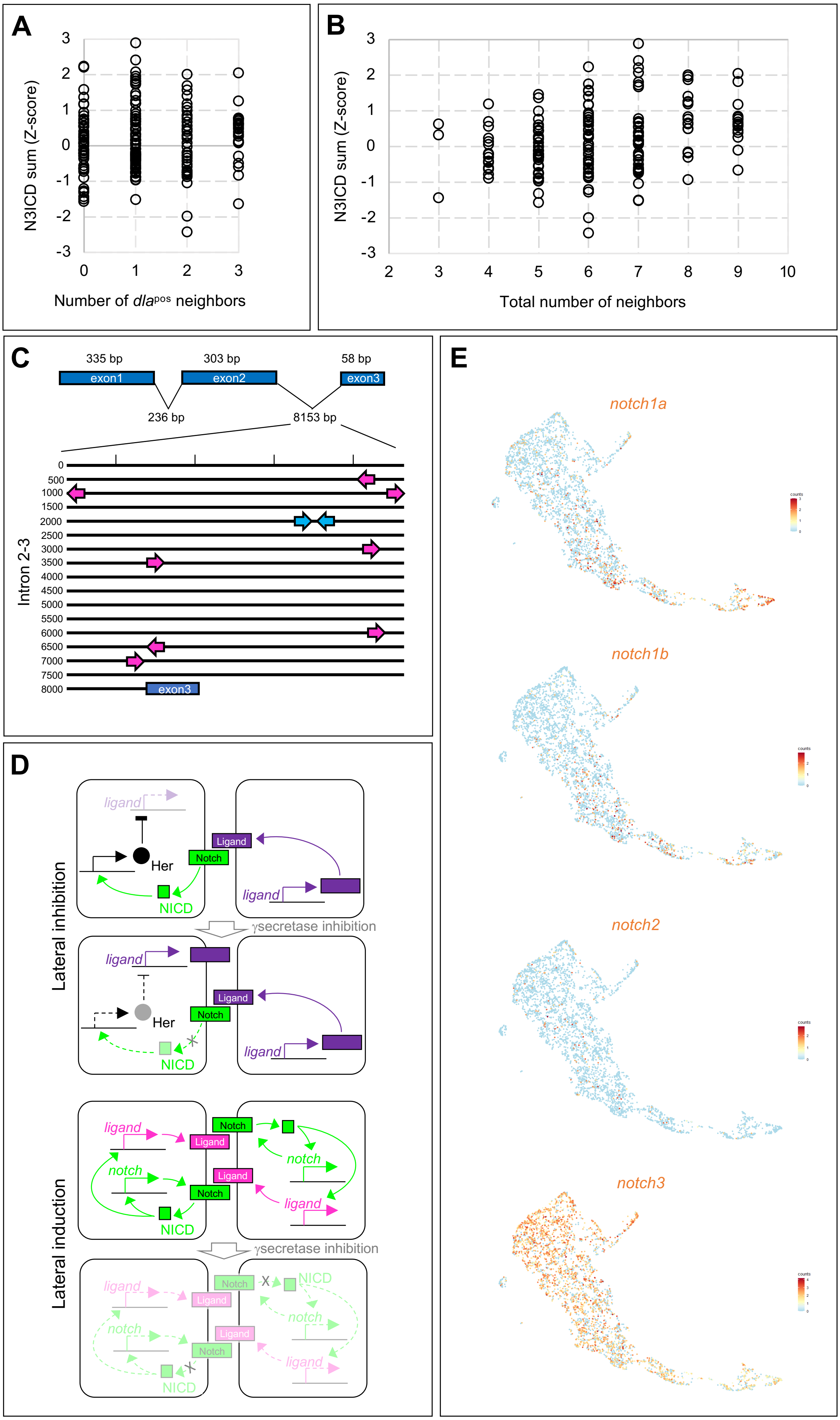
A
N3ICD sum (Z-score)
Number of dla^pos neighbors
B
N3ICD sum (Z-score)
Total number of neighbors
C
335 bp
303 bp
58 bp
exon1
exon2
exon3
236 bp
8153 bp
Intron 2-3
exon3
D
Lateral inhibition
ligand
Her
Notch
Ligand
NICD
γsecretase inhibition
Lateral induction
notch
E
notch1a
notch1b
notch2
notch3

Figure S6. Notch3 signaling in relation with *dla* expression in neighboring cells and with *jag1b* transcription. A,B. Quantification, in *dla*^{neg} cells, of nuclear N3ICD-AG levels as in Fig.4J,K, and classification of each cell according to its number of *dla*^{pos} neighbors (A – Spearman's ρ=0.05) or its total number of neighbors (B – Spearman's ρ=0.36) (Z-score of N3ICD pixel sum; 3 brains, n=174 *dla*^{neg} cells in total and n=180 *dla*^{pos} neighbors - source data are provided as a Source Data file). **C.** Scheme of the *jag1b* locus with position of RPBj binding sites within the regulatory upstream sequence. Single sites are indicated in pink, and a head-to-head site known to be efficiently targeted by NICD [7] is indicted in blue. **D.** Scheme depicting the expected effect of a mild LY411575 treatment (lowering γsecretase activity) on the expression of Notch ligands in the case of lateral inhibition (top, increase of ligand's expression) vs lateral induction (bottom, decrease of ligand's expression). **E.** Expression levels of the different Notch receptor genes, plotted on the scRNAseq UMAP of neural stem and progenitor cells of the adult pallium in WT fish (as in Fig.S5A-G) [5].

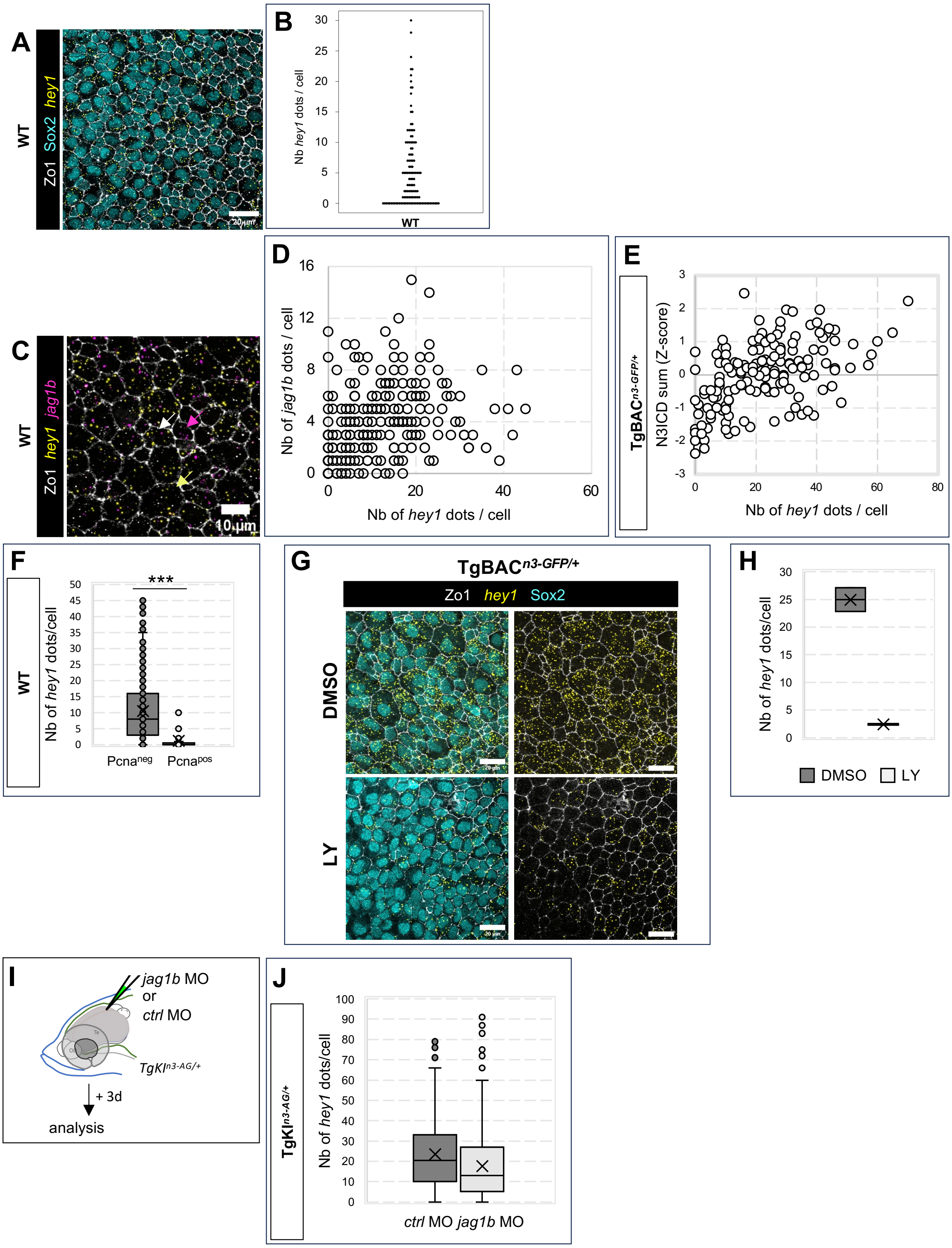

<u>Figure S7.</u> *hey1* expression is sensitive to Jag1b/Notch3 signaling. A. Expression of *hey1* revealed by smFISH (yellow) in a whole-mount adult WT adult pallium (dorsal confocal view) together with IHC for Zo1 (white) and Sox2 (cyan). Scale bar: 20 µm. **B.** Corresponding quantification of *hey1* transcripts (number of smFISH dots per cell; 1 brain, n=118 cells). **C.** Expression of the Notch ligand *jag1b* (magenta) and the Notch target *hey1* (yellow) revealed by smFISH in a whole-mount adult WT adult pallium (dorsal confocal view) together with IHC for Zo1 (white). Some cells express only one player (magenta and yellow arrows), others show co-expression of both (white arrow). Scale bar: 10 µm. **D.** Corresponding scatterplot showing the relative distribution in each cell of (C). There is a weak cell-

autonomous correlation between ligand and target expression (n=218 cells, 1 brain; Spearman's ρ=0.38). **E.** Scatter plot of N3ICD levels (Z-score of N3ICD pixel sum) and *hey1* transcription (smFISH, number of dots per cell) in the adult pallium of a TgBAC$^{n3\text{-}GFP/+}$ fish. There is a direct cell-autonomous correlation between the two parameters (n=153 cells from 2 brains; Spearman's ρ=0.15). **F.** Boxplot of *hey1* expression levels (smFISH, number of dots per cell) in Pcna^{neg} vs Pcna^{pos} cells in WT adult pallia. *hey1* expression is significantly lower in proliferating cells (Wilcoxon rank sum test, p-value=2.053e-09) (n=336 cells from 2 brains). **G.** Expression of *hey1* (smFISH, yellow), together with IHC for Zo1 (white) and Sox2 (cyan) in the pallial ventricular zone of TgBAC$^{n3\text{-}GFP/+}$ adults treated with 10 mM LY-411575 or solvent only (DMSO) for 24h (dorsal whole-mount confocal views). Scale bar: 20 µm. **H.** Corresponding quantification of *hey1* expression levels (mean of number of dots per cell) in DMSO vs LY conditions (DMSO: n=2 hemispheres from 1 brain, LY: n=3 hemispheres from 2 brains). **I.** Schematic of the experiment assessing Jag1 knockdown effect on *hey1*: TgKI$^{n3\text{-}AG/+}$ fish were injected with *jag1bMO* or *ctrlMO* followed by a three-day chase before *hey1* smFISH detection. **J.** Corresponding quantification of *hey1* expression levels (mean of number of dots per cell) in the two MO conditions. A weak decrease of *hey1* expression after 72h downregulation of *jag1b* translation could be observed (*ctrlMO*: n=320 cells from 2 brains, *jag1bMO*: n=321 cells from 2 brains).
Boxplots show median (central line), mean (X), interquartile range (box), whiskers (bars) and outliers (dots). All source data are provided as a Source Data file

Supplementary Table 1

All the reagents, materials and resources used in this study are listed:

REAGENT or RESOURCE	SOURCE	IDENTIFIER
**Antibodies**		
Mouse monoclonal anti-Zrf1 (1:200)	ZIRC	zfin.org/ZDB-ATB-081002-46
Chicken polyclonal anti-GFP (1:500)	Aves Labs	Cat#GFP-1020; RRID:AB_10000240
Rat polyclonal anti-RFP (Chromotek clone 5f8-150) (1:250)	ThermoFisher Scientific	Cat#17609716; RRID:AB_2336064
Rabbit polyclonal anti-Sox2 (1:250)	Abcam	Cat#ab97959; RRID:AB_2341193
Mouse monoclonal anti-PCNA (1:250)	Santa Cruz Biotechnology	Cat# sc-56; RRID:AB_628110
Mouse monoclonal anti-AzamiGreen (1:250)	Clinisciences	Cat#M102-3M; RRID:AB_590583
Rabbit polyclonal anti-AzamiGreen (1:250)	Clinisciences	Cat#PM052M; RRID:AB_2827721
Rat monoclonal anti-BrdU (1:200)	Abcam	Cat#ab6326; RRID:AB_305426
Mouse monoclonal anti-HuC/D (clone 16A11) (1:250)	ThermoFisher Scientific	Cat#A-21271; RRID:AB_221448
Rabbit polyclonal anti-Blbp (1:1000)	Merck	Cat#ABN14; RRID:AB_11210770

Mouse monoclonal anti-ZO1, Alexa Fluor 647 (clone ZO1-1A12) (1:250)	ThermoFisher Scientific	Cat#MA3-39100-A647; RRID:AB_2663167
Mouse monoclonal anti-ZO1, Unconjugated (clone ZO1-1A12) (1:250)	ThermoFisher Scientific	Cat# 33-9100; RRID:AB_87181
Mouse monoclonal anti-ZO1, Alexa Fluor 488 (clone ZO1-1A12) (1:250)	ThermoFisher Scientific	Cat# MA3-39100-A488; RRID:AB_2663169
Rabbit polyclonal anti-Rab5 (S-19) (1:100)	Santa Cruz Biotechnology	Cat#sc-309; RRID:AB_632295
Mouse monoclonal anti-Rab7 (1:100)	Merck	Cat#R8779; RRID:AB_609910
Rabbit polyclonal anti-Rab11 (1:200)	Gentex	Cat#GTX127328; RRID:AB_11171170
Rabbit polyclonal anti-Lamp1 (1:100)	Abcam	Cat#ab24170; RRID:AB_775978
Sheet anti-Digoxigenin (Fab fragment), Peroxydase conjugated (1:200)	Merck	Cat#11207733910; RRID:AB_514500
Goat Anti-Chicken IgG(H+L), Alexa Fluor 488 Conjugated (1:1000)	ThermoFisher Scientific	Cat#A-11039; RRID:AB_142924
Goat anti-Mouse IgG1, Alexa Fluor 488 Conjugated (1:1000)	ThermoFisher Scientific	Cat# A-21121; RRID:AB_2535764
Goat anti-Mouse IgG1, Alexa Fluor 647 Conjugated (1:1000)	ThermoFisher Scientific	Cat# A-21240; RRID:AB_2535809
Goat anti-Mouse IgG2a, Alexa Fluor 488 conjugated (1:1000)	ThermoFisher Scientific	Cat#A-21131; RRID:AB_2535771
Goat anti-Mouse IgG2a, Alexa Fluor 633 conjugated (1:1000)	ThermoFisher Scientific	Cat#A-21136; RRID:AB_2535775
Goat anti-Mouse IgG2b, Alexa Fluor 546 conjugated (1:1000)	ThermoFisher Scientific	Cat# A-21143; RRID:AB_2535779
Goat Anti-Rabbit IgG(H+L), Alexa Fluor 405 Conjugated (1:1000)	ThermoFisher Scientific	Cat#A-31556; RRID:AB_221605
Goat Anti-Rabbit IgG(H+L), Alexa Fluor 488 Conjugated (1:1000)	ThermoFisher Scientific	Cat#A-11008; RRID:AB_143165
Goat Anti-Rabbit IgG(H+L), Alexa Fluor 546 Conjugated (1:1000)	ThermoFisher Scientific	Cat#A-11010; RRID:AB_2534077
Goat anti-Rat IgG(H+L), Alexa Fluor 546 Conjugated (1:1000)	ThermoFisher Scientific	Cat#A-11081; RRID: RRID:AB_2534125
**Chemicals, peptides, and recombinant proteins**		
LY-411575	Merck	Cat#SML0506
5-Bromo-2-deoxyuridine (BrdU)	Merck	Cat#B5002
Paraformaldehyde Aqueous Solution 16%	ThermoFisher Scientific (Electron Microscopy Sciences)	Cat#15710

Antigen Retrevial (HistoVT One)	Nacalai Tesque	Cat#06380-05
PBS 10X	ThermoFisher Scientific	Cat#10649743
Goat Serum Donor Herd	Merck	Cat#G6767
TWEEN 20	Merck	Cat#P9416
Triton-X100	Merck	Cat#X100
Natural mouse serum	Abcam	Cat#ab7486
Petrolatum Jelly	Merck	Cat#16415
Lanolin	Merck	Cat#L7387
Paraffin wax	Merck	Cat# 76242
Aqua-Poly/Mount	Polysciences	Cat#18606
DIG RNA Labeling Mix	Merck	Cat#11277073910
T3 RNA polymerase	Promega	Cat#P2083
T7 RNA polymerase	Promega	Cat#P2075
Cas9 nuclease	Toolgen (Labomics)	Cat#Cas9-TOO-250
Green GoTaq G2 Hot Start Master Mix	Promega	Cat#M7422
DNAse I recombinant	Merck	Cat#4716728001
RNase inhibitor	Takara Bio	Cat#2313A
Proteinase K recombinant	ThermoFisher Scientific	Cat#EO0491
T7 Endonuclease I	NEB	Cat#M0302S
Anti-Digoxygen-AP, Fab fragments	Merck	Cat#11093274910
Hydrogen peroxide solution 30 %	Merck	Cat#216763
Formamide	Merck	Cat#47671
Invitrogen SSC UltraPure, 20X	ThermoFisher Scientific	Cat#10515203
MS-222	ThermoFisher Scientific	Cat#A5040
DAPI	Merck	Cat#d8417
**Critical commercial assays**		
Phire Animal Tissue Direct PCR Kit	ThermoFisher Scientific	Cat#F140WH
StrataClone PCR Cloning Kit	Agilent Technologies	Cat#240205
QIAquick Gel Extraction Kit	Qiagen	Cat#28704
ProbeQuant G-50 Micro Columns	Merck	Cat#GE28-9034-08
RNAscope Multiplex Fluorescent Reagent Kit v2	Bio-Techne	Cat#323100
RNAscope Hiplex probe diluent	Bio-Techne	Cat#324301

OPAL 480 reagent pack	Akoya Biosciences	Cat#FP1500001KT
OPAL 570 reagent pack	Akoya Biosciences	Cat#FP1488001KT
OPAL 650 reagent pack	Akoya Biosciences	Cat#FP1496001KT
**Experimental models: Organisms/strains**		
D. rerio / AB WT strain	N/A	ZDB-GENO-960809-7
D. rerio / *Casper (roy−/−;nacre−/−)*	Zebrafish International ResourceCenter, Eugene, OR	ZDB-FISH-150901-6638
D. rerio / *TgBAC*(*notch3:notch3-GFP-P2A-nlsRFP*)^{ip13Tg}	This study	ZDB-ALT-230810-3
D. rerio / *TgKI(notch3:notch3-mAG-P2A-nlsRFP)*^{ip14Tg}	This study	ZDB-ALT-230814-2
D. rerio / Tg(*dla:GFP*)	Zebrafish International ResourceCenter, Eugene, OR	ZDB-TGCONSTRCT-100823-1
**Oligonucleotides**		
vivo-Morpholino Standard Control Oligo	GeneTools	https://store.gene-tools.com/node/334
MO1-jag1b	GeneTools	https://zfin.org/ZDB-MRPHLNO-041207-3
Alt-R CRISPR-Cas9 crRNA	IDT	Custom
Alt-R CRISPR-Cas9 tracrRNA	IDT	Cat#1072533
Dr-dla-C3 RNAscope probe (targeting 1568-2518 of NM_130954.2)	Bio-Techne	Ref#1133701-C3
Dr-jag1a-C2 RNAscope probe (targeting 1299-2372 of NM_131861.1)	Bio-Techne	Ref#1189971-C2
Dr-jag1b-C2 RNAscope probe (targeting 1129-2256 of NM_131863.2)	Bio-Techne	Ref#1189981-C2
Dr-jag2b-C1 RNAscope probe (targeting 3011-3955 of NM_131862.1)	Bio-Techne	Ref#1039801-C1
Dr-hey1-C1 RNAscope probe (targeting 2-1091 of the sequence NM_212561.1)	Bio-Techne	Ref#1234851-C1
**Recombinant DNA**		
BAC CH211-69E21	Zfin	ZDB-BAC-050218-541
Plasmid pCS2_GFP-P2A-nlsRFP	This study	N/A
Plasmid pCS2_AzamiGreen-P2A-nlsRFP	This study	N/A
pmAG1-MN1 (CoralHue monomeric AzamiGreen)	MBL	Cat#AM-V0033

Software and algorithms		
ZEN software	Carl Zeiss	RRID:SCR_018163 (Black edition); RRID:SCR_013672 (Blue edition)
Imaris	Bitplane	https://www.imaris.oxinst.com/packages; RRID: SCR_007370
Fiji	Fiji	https://fiji.sc; RRID: SCR_002285
R Project for Statistical Computing	Posit Software	RRID:SCR_001905
Napari 0.4.18 (under Python 3.9)	In-house code based on Epyseg [8] and Big-FISH [9]	RRID:SCR_022765

Supplementary References

1. Concordet, J.-P. & Haeussler, M. CRISPOR: intuitive guide selection for CRISPR/Cas9 genome editing experiments and screens. *Nucleic Acids Research* **46**, W242–W245 (2018).
2. Moreno-Mateos, M. A. *et al.* CRISPRscan: designing highly efficient sgRNAs for CRISPR-Cas9 targeting in vivo. *Nat Methods* **12**, 982–988 (2015).
3. Couturier, L., Trylinski, M., Mazouni, K., Darnet, L. & Schweisguth, F. A fluorescent tagging approach in Drosophila reveals late endosomal trafficking of Notch and Sanpodo. *J Cell Biol* **207**, 351–363 (2014).
4. Couturier, L., Vodovar, N. & Schweisguth, F. Endocytosis by Numb breaks Notch symmetry at cytokinesis. *Nat Cell Biol* **14**, 131–139 (2012).
5. Morizet, D., Foucher, I., Alunni, A. & Bally-Cuif, L. Reconstruction of macroglia and adult neurogenesis evolution through cross-species single-cell transcriptomic analyses. *Nat Commun* **15**, 3306 (2024).
6. Mancini, L. *et al.* Apical size and deltaA expression predict adult neural stem cell decisions along lineage progression. eadg7519 (2023).
7. Arnett, K. L. *et al.* Structural and mechanistic insights into cooperative assembly of dimeric Notch transcription complexes. *Nat Struct Mol Biol* **17**, 1312–1317 (2010).
8. Aigouy, B. & Prud'homme, B. Segmentation and Quantitative Analysis of Epithelial Tissues. *Methods Mol Biol* **2540**, 387–399 (2022).
9. Imbert, A. *et al.* FISH-quant v2: a scalable and modular analysis tool for smFISH image analysis. 2021.07.20.453024 Preprint at https://doi.org/10.1101/2021.07.20.453024 (2021).
