# Supplementary material for: Jagged-mediated lateral induction patterns Notch3 signaling within adult neural stem cell populations
Source: Nat Commun. 2026 Mar 14;17:3986. doi: 10.1038/s41467-026-70478-0 (PMC13136484; doi:10.1038/s41467-026-70478-0)
Supplement: Supplementary file 2 — Reporting Summary [file 41467_2026_70478_MOESM2_ESM.pdf]

nature portfolio

Corresponding author(s): Laure Bally-Cuif

Last updated by author(s): 2026/01/14

Reporting Summary

Nature Portfolio wishes to improve the reproducibility of the work that we publish. This form provides structure for consistency and transparency in reporting. For further information on Nature Portfolio policies, see our Editorial Policies and the Editorial Policy Checklist.

Statistics

For all statistical analyses, confirm that the following items are present in the figure legend, table legend, main text, or Methods section.

n/a	Confirmed	
☐	☒	The exact sample size (*n*) for each experimental group/condition, given as a discrete number and unit of measurement
☐	☒	A statement on whether measurements were taken from distinct samples or whether the same sample was measured repeatedly
☐	☒	The statistical test(s) used AND whether they are one- or two-sided<br>*Only common tests should be described solely by name; describe more complex techniques in the Methods section.*
☐	☒	A description of all covariates tested
☐	☒	A description of any assumptions or corrections, such as tests of normality and adjustment for multiple comparisons
☐	☒	A full description of the statistical parameters including central tendency (e.g. means) or other basic estimates (e.g. regression coefficient) AND variation (e.g. standard deviation) or associated estimates of uncertainty (e.g. confidence intervals)
☐	☒	For null hypothesis testing, the test statistic (e.g. *F*, *t*, *r*) with confidence intervals, effect sizes, degrees of freedom and *P* value noted<br>*Give P values as exact values whenever suitable.*
☐	☒	For Bayesian analysis, information on the choice of priors and Markov chain Monte Carlo settings
☐	☒	For hierarchical and complex designs, identification of the appropriate level for tests and full reporting of outcomes
☒	☐	Estimates of effect sizes (e.g. Cohen's *d*, Pearson's *r*), indicating how they were calculated

Our web collection on statistics for biologists contains articles on many of the points above.

Software and code

Policy information about availability of computer code

Data collection	Data were produced during the study without requiring any custom code.
Data analysis	All data (raw and processed) can be found in Zenodo at https://zenodo.org/records/18244815

For manuscripts utilizing custom algorithms or software that are central to the research but not yet described in published literature, software must be made available to editors and reviewers. We strongly encourage code deposition in a community repository (e.g. GitHub). See the Nature Portfolio guidelines for submitting code & software for further information.

Data

Policy information about availability of data

All manuscripts must include a data availability statement. This statement should provide the following information, where applicable:

- Accession codes, unique identifiers, or web links for publicly available datasets
- A description of any restrictions on data availability
- For clinical datasets or third party data, please ensure that the statement adheres to our policy

All data (raw and processed) can be found in Zenodo at https://zenodo.org/records/18244815

Research involving human participants, their data, or biological material

Policy information about studies with human participants or human data. See also policy information about sex, gender (identity/presentation), and sexual orientation and race, ethnicity and racism.

Reporting on sex and gender	NR (no human participants or data)
Reporting on race, ethnicity, or other socially relevant groupings	NR (no human participants or data)
Population characteristics	NR (no human participants or data)
Recruitment	NR (no human participants or data)
Ethics oversight	NR (no human participants or data)

Note that full information on the approval of the study protocol must also be provided in the manuscript.

Field-specific reporting

Please select the one below that is the best fit for your research. If you are not sure, read the appropriate sections before making your selection.

☒ Life sciences ☐ Behavioural & social sciences ☐ Ecological, evolutionary & environmental sciences

For a reference copy of the document with all sections, see nature.com/documents/nr-reporting-summary-flat.pdf

Life sciences study design

All studies must disclose on these points even when the disclosure is negative.

Sample size	No precise sample sizes were calculated. The number of fish used for each experiment took into account our goal to be able to identify, in the worst case, a cell population (proliferating cells) representing roughly 5% of the neural stem cell pool, and our own previous analyses over the last >15 years.
Data exclusions	There were no data exclusion, all plots can be reproduced using the full set of raw data provided in Data Source file
Replication	The number of biological and technical replicates, as appropriate, is indicated for each analysis (each relevant figure panel and/or its legend).
Randomization	Fish were selected only on the basis of their age to focus on adult neurogenesis and on whether they expressed relevant transgenes. They were selected at random among suitable fish in our animal facility with no exclusion criteria based on gender. No other specific parameters were taken into account when selecting fish for experiments.
Blinding	There was no blinding performed for the quantifications but samples were blinded for the modelling and analyses were conducted by two different persons.

Reporting for specific materials, systems and methods

We require information from authors about some types of materials, experimental systems and methods used in many studies. Here, indicate whether each material, system or method listed is relevant to your study. If you are not sure if a list item applies to your research, read the appropriate section before selecting a response.

Materials & experimental systems

n/a	Involved in the study	
☐	☒	Antibodies
☒	☐	Eukaryotic cell lines
☒	☐	Palaeontology and archaeology
☐	☒	Animals and other organisms
☒	☐	Clinical data
☒	☐	Dual use research of concern
☒	☐	Plants

Methods

n/a	Involved in the study	
☒	☐	ChIP-seq
☒	☐	Flow cytometry
☒	☐	MRI-based neuroimaging

Antibodies

Antibodies used	The 28 different antibodies (primary or secondary) used in this study are listed in the Materials table (Supplementary data)
Validation	These antibodies have been used in hundreds of publications. See for example Than-Trong et al. 2020 and Mancini et al, Sci. Adv. 2023 doi: 10.1126/sciadv.adg7519

Animals and other research organisms

Policy information about studies involving animals; ARRIVE guidelines recommended for reporting animal research, and Sex and Gender in Research

Laboratory animals	D.Rerio, AB strain, 3 months post-fertilization.
Wild animals	No wild animals were used in this study.
Reporting on sex	Mixed
Field-collected samples	No field-collected samples were used in this study.
Ethics oversight	All procedures relating to zebrafish (Danio rerio) care and treatment conformed to the directive 2010/63/EU of the European Parliament and of the council of the European Union. The animal study protocol was approved by the Ethics Committee n°39 of Institut Pasteur (authorization #36936, April 26th, 2022) and DDPP-2021-021 of the Direction Départementale de la Protection des Populations de Paris.

Note that full information on the approval of the study protocol must also be provided in the manuscript.

Plants

Seed stocks	*Report on the source of all seed stocks or other plant material used. If applicable, state the seed stock centre and catalogue number. If plant specimens were collected from the field, describe the collection location, date and sampling procedures.*
Novel plant genotypes	*Describe the methods by which all novel plant genotypes were produced. This includes those generated by transgenic approaches, gene editing, chemical/radiation-based mutagenesis and hybridization. For transgenic lines, describe the transformation method, the number of independent lines analyzed and the generation upon which experiments were performed. For gene-edited lines, describe the editor used, the endogenous sequence targeted for editing, the targeting guide RNA sequence (if applicable) and how the editor was applied.*
Authentication	*Describe any authentication procedures for each seed stock used or novel genotype generated. Describe any experiments used to assess the effect of a mutation and, where applicable, how potential secondary effects (e.g. second site T-DNA insertions, mosiacism, off-target gene editing) were examined.*
